# Global trends in dengue research in veterinary medicine (2019–2024): A bibliometric analysis of scientific output, collaborations, and thematic shifts

**DOI:** 10.14202/vetworld.2025.2311-2319

**Published:** 2025-08-14

**Authors:** Fran Espinoza-Carhuancho, Cesar Mauricio-Vilchez, Lucia Quispe-Tasayco, Frank Mayta-Tovalino

**Affiliations:** 1 Bibliometrics, Evidence Assessment and Systematic Reviews Group (BEERS), Faculty of Medicine, Universidad Científica del Sur, Lima 15058, Peru; 2 Department of Academic, Research, Innovation and Entrepreneurship Unit, Universidad Nacional Federico Villarreal, Lima 15058, Peru; 3 Vice-Rectorate for Research, Universidad San Ignacio de Loyola, Lima 12175, Peru

**Keywords:** *Aedes aegypti*, bibliometrics, dengue virus, international collaboration, mosquito-borne diseases, One Health, vector control, veterinary medicine

## Abstract

**Background and Aim:**

Dengue is a significant mosquito-borne viral disease with increasing public health relevance, particularly in tropical and subtropical regions. Although it primarily affects humans, veterinary research plays a crucial role in understanding disease dynamics, particularly through the use of animal models and vector control strategies. This study aimed to analyze global research trends on dengue within the field of veterinary medicine between 2019 and 2024, identifying patterns in productivity, collaboration, and emerging thematic priorities.

**Materials and Methods:**

A bibliometric analysis was conducted using Scopus-indexed publications retrieved on December 15, 2024. The search included terms related to dengue and *Aedes* species within the veterinary subject area. Data were analyzed using SciVal for metrics and collaboration patterns and RStudio for statistical summaries and visua-lizations. Inclusion criteria encompassed peer-reviewed studies on dengue relevant to veterinary contexts published between 2019 and 2024.

**Results:**

A total of 742 publications were identified across 67 journals, including 652 articles and 60 reviews. Annual output showed a 3.01% decline, with an average document age of 2.56 years and 9.0 citations per article. International collaboration was evident in 41.51% of publications, with Brazil, Argentina, and Australia emerging as key contributors. Thematic evolution demonstrated a shift from basic entomological studies (2019–2020) to applied control strategies, including *Wolbachia*-based mosquito interventions and citizen surveillance (2021–2022). The most prolific institutions were Fundação Oswaldo Cruz (Brazil) and the University of Florida (USA), while the journal Parasites and Vectors had the highest publication volume.

**Conclusion:**

This bibliometric review highlights the growing interdisciplinary nature of dengue research in veterinary medicine and the pivotal role of international collaboration. The observed decline in publication rate underscores the need to reinvigorate research efforts. The findings advocate for a One Health approach integrating veterinary, ecological, and public health disciplines to address vector-borne diseases. Future efforts should focus on enhancing global partnerships, standardizing animal models, and supporting innovative vector control strategies to mitigate the burden of dengue.

## INTRODUCTION

Dengue is a mosquito-borne viral disease of significant global concern, particularly in tropical and subtropical regions. Although animals are not infected with dengue in the same way as humans, veterinary science plays a crucial role in studying the pathogenesis of dengue virus (DENV) through the use of animal models. These models are crucial for testing hypoth-eses that are either ethically or technically infeasible in human subjects. They contribute valuable insights into virus–host interactions and disease progression, supporting the development of antivirals, vaccines, and dengue-related therapeutics [1].

The growing global burden of dengue has been attributed to multiple factors, including population growth, urban density, unregulated urbanization, and climate change. Over the past two decades, the incidence of dengue has escalated, exacerbated by decreasing access to safe drinking water and ineffective vector control measures [2]. At present, nearly 70% of the global dengue burden is concentrated in Southeast Asia and the Western Pacific regions [3, 4].

DENV belongs to the genus *Flavivirus* within the family *Flaviviridae* and comprises four antigenically distinct serotypes. These serotypes circulate cyclically in endemic areas, each eliciting unique immune respo-nses despite genetic similarity [5, 6]. The primary vec-tors, *Aedes aegypti* and *Aedes albopictus*, are highly adaptable, breeding in stagnant water and exhibiting peak activity during daylight hours. Their lifecycle, com-prising egg, larva, pupa, and adult stages, is typically completed within 10 days [7].

Infection with a single dengue serotype can confer lifelong immunity against that strain; however, subsequent infection by a different serotype increases the risk of severe disease due to antibody-dependent enhancement. Clinical manifestations in humans range from mild febrile illness to severe dengue hemo-rrhagic fever, characterized by plasma leakage, organ dysfunction, and potential fatality. Furthermore, mos-quito-derived factors, including salivary proteins, can exacerbate disease severity by influencing host immune responses [8].

The absence of a universally accepted animal model remains a barrier to fully elucidating dengue immunopathogenesis. Nonetheless, experimental mod-els using mice, pigs, and birds have provided critical insights into disease mechanisms and intervention strategies. Although mice are not naturally susceptible, certain laboratory conditions allow productive infection, contributing to vaccine and drug development [9, 10].

The relevance of dengue to veterinary medicine is anchored in the indispensable role of animal mod-els for pre-clinical research. While dengue does not cause disease in animals, veterinary-based studies offer mechanistic insights that human studies alone cannot provide. This highlights the importance of a One Health approach, which integrates human, animal, and enviro-nmental health perspectives to combat mosquito-borne diseases [1–5].

This bibliometric analysis offers a comprehensive overview of dengue-related research in veterinary medicine from 2019 to 2024. It emphasizes patterns in scientific productivity, international collaboration, and thematic evolution. By mapping research trends and emerging priorities, this study supports evidence-informed strategies for vector control and zoonotic disease prevention. Its novelty lies in framing dengue as a veterinary as well as a public health concern, highlighting the critical intersection of entomology, animal models, and global health. Findings are expected to guide future research, policy development, and cross-sectoral collaboration for more effective surveillance and intervention strategies [2-13].

Despite the growing global concern surrounding DENV and its public health implications, most scie-ntific literature and surveillance efforts remain heavily concentrated on human clinical outcomes, epide-miology, and virology. Consequently, the veterinary dimension of dengue research remains underexplored and fragmented. While animal models are routinely employed in experimental dengue studies – particularly in evaluating vaccines, immunopathogenesis, and antiviral therapies – there is a paucity of consolidated information analyzing the scope, trends, and collab-oration networks in this domain. Moreover, the current bibliometric studies on dengue tend to focus on clinical or public health disciplines, often neglecting contributions made by veterinary science, especially in areas such as vector-host interaction, experimental virology, and One Health approaches. This lack of a comprehensive synthesis limits the ability to identify strategic resea-rch priorities and weakens interdisciplinary responses to mosquito-borne diseases. Furthermore, although international collaborations have proven effective in enhancing dengue surveillance and control, little is known about their extent or structure within the vete-rinary research landscape.

The present study aims to conduct a compre-hensive bibliometric analysis of global dengue research within the field of veterinary medicine from 2019 to 2024. Specifically, it seeks to (1) quantify scientific out-put and examine publication trends; (2) identify leading institutions, authors, and journals in veterinary-related dengue studies; (3) assess international research colla-borations; and (4) explore thematic evolution and priority research areas within this interdisciplinary field. By mapping the research landscape, the study intends to highlight underrepresented topics, foster interdisciplinary dialog, and promote the integration of veterinary science into global dengue prevention and control strategies. Ultimately, the findings will inform future research directions and policy frameworks alig-ned with the One Health paradigm.

## MATERIALS AND METHODS

### Ethical approval

As this study did not involve any experimen- tation on animals or human participants, ethical approval was not required. The study adopted a bibliometric design to analyze publication trends, sci- entific productivity, and thematic developments in dengue-related veterinary research published between 2019 and December 2024. This study follow- ed Reporting and Measurement of Items for Bibliome-tric or Scientometric Studies (RAMIBS) guidelines [14].

### Study period and location

The study was conducted in December 2024 at the Library of the Universidad San Ignacio de Loyola (Lima, Peru).

### Literature search strategy

A comprehensive literature search was conducted on December 15, 2024, using the Scopus database (Els-evier, Netherlands). The following search formula was employed to identify relevant publications:

TITLE-ABS (“Dengue” OR “Dengue Fever” OR “Dengue Virus” OR “Dengue Disease” OR “DENV” OR “Dengue Infection” OR “Mosquito-Borne Viral Disease” OR “*Aedes aegypti*” OR “*Aedes albopictus*” OR “Dengue Hemorrhagic Fever”) AND SUBJAREA (vete) AND PUBY-EAR >2018 AND PUBYEAR <2025.

This search yielded a total of 742 documents, including 652 original articles, 60 reviews, 12 errata, 6 notes, 6 conference proceedings, 2 letters, 2 editorials, 1 short survey, and 1 book chapter.

### Study design and eligibility criteria

The study adopted a bibliometric design to analyze publication trends, scientific productivity, and thematic developments in dengue-related veterinary research published between 2019 and December 2024.

### Inclusion criteria

- Publications focusing on dengue research relevant to veterinary science
- Indexed in Scopus
- Published between January 01, 2019, and December 15, 2024
- All publication types (e.g., original articles, reviews, conference papers, and book chapters).

### Exclusion criteria

- Publications unrelated to the veterinary aspects of dengue
- Articles outside the specified publication window
- Non-peer-reviewed literature.
- This approach ensured the representativeness, rel-iability, and reproducibility of the dataset for analysis.

### Data analysis tools and software

- SciVal (Elsevier, Netherlands) was utilized for its robust analytical capabilities, including:
- Field-Weighted Citation Impact (FWCI)
- International collaboration metrics
- Source and author-level performance indicators.

R Studio R version 4.5.0 (2025-04-11 UCRT, R Foundation, Vienna, Austria) was employed for adva-nced statistical analysis and visualization. Its flexibility, along with access to specialized packages, enabled cust-omized bibliometric mapping, graphical outputs, and temporal trend analyses.

### Bibliometric parameters and analytical approach

Multiple bibliometric indicators were computed to assess the scientific landscape of dengue research in veterinary medicine:

- Annual publication growth rate
- Mean document age
- Average citations per publication
- Reference count
- International collaboration frequency.

The analysis also applied Lotka’s Law to assess aut-hor productivity patterns and Bradford’s Law to evaluate core journal dispersion. To examine shifts in research priorities, a thematic evolution analysis was conducted acr-oss defined time intervals. This comprehensive approach enabled both quantitative and qualitative insights into the development of the field over time.

## RESULTS

### Scientific output and publication characteristics (2019–2024)

Between 2019 and 2024, a total of 742 documents from 67 different sources were identified. These included 652 original articles, 60 reviews, 12 errata, 6 notes, 6 conference papers, 2 editorials, 2 letters, 1 short survey, and 1 book chapter. The publication trend showed an annual decline of 3.01%, and the average age of the documents was 2.56 years. On average, each publication received 9.0 citations, contributing to a total of 33,679 references. The authorship analysis revealed that 4299 researchers contributed to the dataset, with a mean of 7.36 co-authors per paper, and 41.51% of the documents involved international collaboration (Table 1).

**Table 1:** Main characteristics.

Description	Results
Timespan	2019–2024
Sources	67
Documents	742
Annual growth (%)	−3.01
Average year of documents	2.56
Average citation counts per document	9.0
References	33,679
Author’s keywords	2,107
Authors	4,299
Authors of single-authored documents	12
Single-authored documents	12
Co-authors per documents	7.3
International co-authorships (%)	41.5
Article	652
Book chapter	1
Conference paper	6
Editorial	2
Letter	2
Note	6
Review	60

### Institutional contributions and citation impact

The analysis of the top ten institutions revealed significant disparities in productivity and impact.

- Fundação Oswaldo Cruz (Brazil) had the highest number of publications (35 documents)
- The University of Florida (USA) had the highest average citations per publication (12.5)
- The Centers for Disease Control and Prevention (CDC) registered the highest FWCI at 2.66.
- Meanwhile, the Centre National de la Recherche Scientifique (France) recorded the highest views per publication (36 views).

These institutions demonstrated leadership in dengue-related veterinary research through both scholarly volume and impact (Table 2).

**Table 2:** Top 10 most productive institutions.

Institution	Country	Scholarly output	Citations per publication	Impact of field-weighted citation	Views per publication
Fundação Oswaldo Cruz	Brazil	35	7.1	2.26	27.8
University of Florida	United States	25	12.5	1.58	17.5
Centre National de la Recherche Scientifique	France	22	8.8	1.68	36
Consejo Nacional de Investigaciones Científicas y Técnicas	Argentina	22	5.1	1.36	18.5
Institut de Recherche Pour le Développement	France	22	9.9	1.51	25.4
Université de Montpellier	France	19	9.5	2.21	28.8
Liverpool School of Tropical Medicine	United Kingdom	18	10.8	2.35	20.3
Centers for Disease Control and Prevention	United States	17	17.9	2.66	27
London School of Hygiene and Tropical Medicine	United Kingdom	16	10.3	2.29	30.5
Universidade de São Paulo, Brazil	Brazil	15	11.8	1.36	32.6

### Most active scientific journals

Among the journals, Parasites and Vectors emerged as the most prolific source with 250 publications.

- Acta Tropica had the highest citations per article (13.3)
- Vaccine recorded the highest CiteScore (2023) among the listed journals.

These journals exemplify both scientific produ-ctivity and influence within the field of vector-borne and veterinary medicine research (Table 3).

**Table 3:** Top 10 most active scientific journals.

Scopus source (Journal)	Scholarly output	Citations per publication	CiteScore 2023	Views per publication
Parasites and Vectors	250	9.6	6.3	27.9
Journal of Medical Entomology	104	10	4.6	16.6
Acta Tropica	72	13.3	5.4	35.7
Vaccine	60	11.3	8.7	21.5
Medical and Veterinary Entomology	54	7.4	3.7	15.6
Parasitology Research	26	3.7	4.1	13.6
Current Research on Parasitology and Vector-Borne Diseases	15	7.1	3.6	7.1
Parasite	14	14.1	5.5	27.1
Transboundary and Emerging Diseases	11	9	8.9	29.7
Veterinary World	11	5.2	3.6	16.5

### Top-contributing authors

The most prolific and highly cited authors in the field were:

- Jérémy Bouyer (International Atomic Energy Age-ncy, Austria) – 10 publications, 14.9 citations/arti-cle, h-index: 37,149 total citations.
- Francis Schaffner (Switzerland) – 8 publications, 18.6 citations/article, h-index: 43,149 citations.
- Kyran M. Staunton (Australia) – 9 publications, 12.6 citations/article.
- Scott Alex Ritchie (Australia) – 8 publications, 14.6 citations/article, h-index: 60.

These metrics indicate both consistent scientific output and strong academic influence in the domain (Table 4).

**Table 4:** Top 10 most important authors.

Authors	Affiliation	Country	Scholarly output	Citations per publication	h-index	Citation count
Bouyer, Jérémy	International Atomic Energy Agency	Austria	10	14.9	37	149
Martins, Ademir J.	Fundação Oswaldo Cruz,	Brazil	10	6.6	32	66
Staunton, Kyran M.	James Cook University, Brisbane, Queensland	Australia	9	12.6	17	113
Ritchie, Scott Alex	Monash University	Australia	8	14.6	60	117
Schaffner, Francis	Francis Schaffner Consultancy	Switzerland	8	18.6	43	149
Lenhart, Audrey E.	Centers for Disease Control and Prevention	United States	7	16	30	112
Lima, José Bento Pereira	Fundação Oswaldo Cruz,	Brazil	7	7.6	33	53
Alto, Barry W. Wilmer	University of Florida	United States	6	7.8	29	47
Maiga, Hamidou	Center national de recherche scientifique et technologique, Burkina Faso, country	Burkina Faso	6	18	17	108
Mamaï, Wadaka	International Atomic Energy Agency	Austria	6	17.2	15	103

### Core journal distribution: Bradford’s law

**Figure 1:**
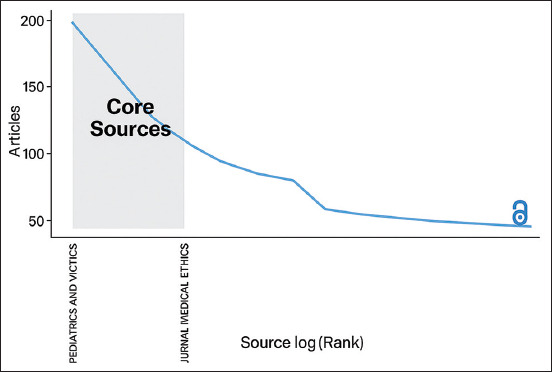
Core sources.

As illustrated in Figure 1, the application of Bradford’s Law classified journal contributions into three productivity zones:

- Zone 1 included high-output journals such as Parasites and Vectors (230 articles) and *Journal of Medical Entomology* (104 articles)
- Zone 2 consisted of moderately productive journals such as Acta Tropica (72 articles) and Vaccine (60 articles)
- Zone 3 included a broad range of journals with fewer than 26 articles each, such as Parasitology Research and Veterinary World.

This pattern confirms that a small number of journals account for the majority of published work, while numerous others contribute to a lesser extent.

### Author productivity pattern: Lotka’s law

**Figure 2:**
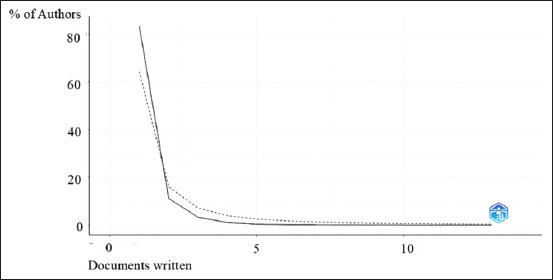
Author productivity.

As shown in Figure 2, Lotka’s Law was applied to evaluate author productivity.

- A vast majority of contributors (3583 authors; 83.3%) published only one paper
- 480 authors (11.2%) contributed two publications
- The frequency of contribution declined sharply bey-ond two papers, with only 47 authors publishing four articles, and just 1 author reaching 13 publications.

This illustrates a typical skewed distribution in which a small number of researchers produce the majo-rity of outputs.

### Thematic evolution of research focus

**Figure 3:**
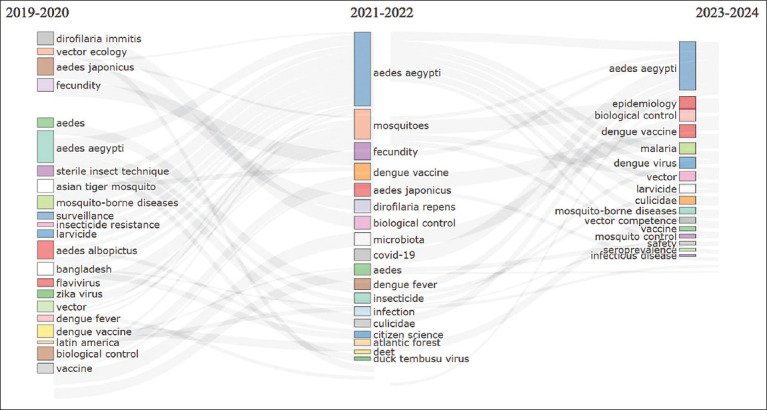
Thematic evolution.

Figure 3 reveals significant thematic shifts in dengue-related veterinary research:

- During 2019–2020, research emphasized the biology and ecology of *A. aegypti* and its role in transmitting dengue, Zika, and chikungunya
- In 2021–2022, the focus transitioned toward vector control strategies, including *Wolbachia*-based interventions, insect sterilization, and citizen surveillance programs.

These changes highlight a growing emphasis on sust-ainable, integrated approaches to vector management in response to emerging public health demands.

### Patterns of international collaboration

**Figure 4:**
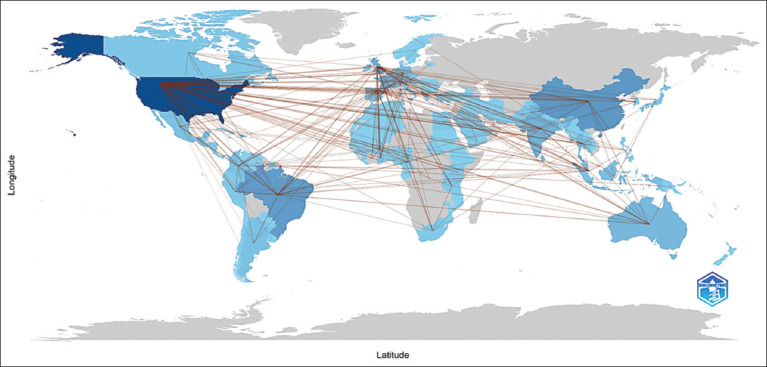
Country collaboration map.

As depicted in Figure 4, Brazil emerged as a central node in international dengue research collaborations:

- Brazil partnered most frequently with Argentina and Peru (5 collaborations each)
- It also maintained strong ties with Australia, Colombia, and Germany (4 collaborations each)
- Argentina collaborated closely with Colombia (3 collaborations)
- Australia engaged with both Guinea and Papua New Guinea in 3 collaborations each.

These findings highlight the importance of intern-ational academic partnerships in advancing dengue res-earch and control efforts.

## DISCUSSION

### Urban animal density and risk of transmission

The high density of animals in urban settings may indirectly increase the risk of human exposure to the DENV by supporting mosquito vector prolife-ration and feeding dynamics. Notably, species such as *A. albopictus*, *A. aegypti*, and *Aedes vittatus*, all prev-alent in the Americas, have been identified as major vectors feeding on a variety of vertebrate hosts [15, 16]. The cohabitation of animal and human populations in urbanized environments provides conditions conducive to increased vector-host interactions, potentially faci-litating disease spread.

### One Health considerations in the context of pandemics

Dengue continues to pose a complex global public health threat, now further exacerbated by the COVID-19 pandemic. The simultaneous circulation of multiple infectious diseases has introduced additional complications, including co-infections and overlapping clinical presentations. These challenges underscore the urgent need for a holistic One Health approach that integrates veterinary, medical, and environmental disc-iplines to address zoonotic and vector-borne threats effectively [17].

### Reservoirs and animal hosts in dengue ecology

Recent studies have identified DENV nucleic acids or antibodies in neotropical mammals such as bats, suggesting potential seropositivity in certain wild species. Non-human primates serve as essential models for investigating dengue pathogenesis and for testing pharmaceutical interventions, as they represent species with documented natural infec- tions. However, the extent to which these animals act as true amplifying reservoirs for DENV transmission remains uncertain due to methodological limitations in existing studies [18].

### Challenges in vector control and environmental dynamics

Controlling *Aedes* mosquito populations – partic-ularly in tropical and subtropical regions – remains a critical challenge for public health systems. A thorough understanding of mosquito biology, ecology, and dise-ase transmission dynamics is necessary to implement successful control strategies. Beyond dengue, *Aedes* species serve as vectors for a variety of pathogens that impact both humans and animals, amplifying their relevance in One Health surveillance. Environmental factors and animal health status are both fundamental to understanding the emergence and propagation of vector-borne diseases. Veterinary research plays an essential role in elucidating these interconnections and in developing interventions to mitigate transmission risks [19, 20].

### Insights from global dengue research trends

A global bibliometric analysis by Liu *et al*. [21] covering 1995–2023 examined 10,767 dengue-related papers, identifying 2021 as the peak year with 747 publications. Their study highlighted substantial pro-gress in virology, vector ecology, and therapeutic inno-vation, including advances in vaccine development (e.g., Dengvaxia, Qdenga, TV003) and biocontrol met-hods, such as *Wolbachia*-infected mosquitoes and gene-editing technologies. The journal PLOS Neglected Tropical Diseases emerged as a leading publication pla-tform in this domain.

### Geographic distribution and climate considerations

Liu *et al*. [22] conducted an analysis spanning lite-rature from 1950 onward, revealing that a vast majority of dengue studies (94.3%) referenced subtropical and tropical countries. The most frequently cited study – authored by Bhatt *et al*. [23] – has received 2604 citations since 2013 and provides pivotal data on the global epidemiology of dengue. These findings high-light a geographic concentration of dengue research in climate-vulnerable regions, although transmission may not be strictly limited to such zones.

### Regional trends in Southeast Asia

According to Maula *et al*. [24], Indonesia has exp-erienced significant growth in dengue-related scientific output over the past decade. Indonesian publications accounted for 5.90% of the 1625 total studies in Southeast Asia but recorded the region’s highest growth rate (28.87%). These publications largely focused on insect vectors and diagnostics, with a recent pivot toward disease outbreaks and vaccine development. The authors emphasized the need for improved rese-arch quality and called for a national roadmap to guide future dengue research in the country.

### Implications for research and policy

The present study contributes to the growing call for a multidisciplinary and collaborative approach to dengue research. By identifying thematic shifts, leading contributors, and research gaps, it supports the form-ulation of strategic public health policies. Applying a One Health framework that merges veterinary, ecological, and medical perspectives is essential for enhancing surveillance systems, improving vector con-trol, and ultimately reducing disease transmission. The bibliometric approach used in this study offers a valuable lens through which to assess research evolution and guide future initiatives.

### Limitations of the study

Despite its strengths, several limitations should be acknowledged. First, the bibliometric dataset was limited to the Scopus database, potentially omitting relevant studies indexed in other repositories such as Web of Science, PubMed, or Embase. Second, the ana-lysis was restricted to veterinary-focused literature, which may have led to the underrepresentation of interdisciplinary research crossing into medical or ecological domains. Third, the evaluation of animal models in dengue research remains complex due to heterogeneous experimental designs and the lack of standardized model protocols. Nevertheless, the study followed RAMIBS guidelines, which enhance reporting quality in bibliometric research, and Scopus’s extensive indexing ensured a sufficiently comprehensive dataset for global trend analysis.

## CONCLUSION

This bibliometric study provides a comprehensive evaluation of global dengue research within the field of veterinary medicine from 2019 to 2024. A total of 742 documents from 67 sources were analyzed, revealing a modest decline in annual scientific output (−3.01%) despite increasing international collaboration (41.51%). Notable institutions such as Fundação Oswaldo Cruz (Brazil), the University of Florida (USA), and the CDC demonstrated high productivity and impact. The most prolific journals included Parasites and Vectors, Acta Tropica, and Vaccine, reflecting the growing integration of vector biology and vaccine development in veterinary dengue research. Thematic analysis indicated a clear transition from ecological studies of *Aedes* mosquitoes to more applied control strategies, including *Wolbachia* deployment and citizen surveillance efforts.

From a practical standpoint, the study undersc-ores the essential role of veterinary science in dengue surveillance, experimental modeling, and vector ecology. The findings support the implementation of One Health strategies that bridge veterinary, human, and environmental health to manage mosquito-borne disease threats more effectively. This is particularly relevant given the complex interplay between animal reservoirs, vector biology, and urban environmental factors.

A key strength of this study lies in its use of dual platforms – SciVal and RStudio – to conduct both quantitative and qualitative analyses, alongside adh-erence to RAMIBS reporting standards, enhancing the credibility and reproducibility of the findings. In addition, the application of bibliometric laws (Lotka’s and Bradford’s) provided insight into author productivity patterns and core publication sources in this niche field.

Nevertheless, the study is limited by its exclusive reliance on the Scopus database, which may not capture relevant publications indexed elsewhere (e.g., Web of Science and PubMed). Moreover, challenges remain in standardizing animal models for dengue research and accurately measuring their impact.

Looking forward, future research should expand to include multidisciplinary datasets and explore dee-per links between veterinary entomology, immunology, and zoonotic disease modeling. It is also imperative to develop standardized experimental frameworks to enhance the utility of animal models in preclinical dengue research.

In conclusion, this bibliometric analysis highlights the evolving landscape of veterinary-based dengue research and its critical role in informing global vec-tor control strategies. To effectively address emerging dengue threats, increased interdisciplinary collab-oration, investment in veterinary public health infras-tructure, and alignment with One Health frameworks are essential. These integrated efforts will strengthen global preparedness and response to mosquito-borne diseases affecting both human and animal populations.

## AUTHORS’ CONTRIBUTIONS

FEC, CMV, FMT, and LQT: Conception of the study. FMT and FEC: Extracted, verified, and analyzed the data and drafted and revised the manuscript. All authors have read, reviewed, criticized, and approved the final manuscript.
